# Malaria after international travel: a GeoSentinel analysis, 2003–2016

**DOI:** 10.1186/s12936-017-1936-3

**Published:** 2017-07-20

**Authors:** Kristina M. Angelo, Michael Libman, Eric Caumes, Davidson H. Hamer, Kevin C. Kain, Karin Leder, Martin P. Grobusch, Stefan H. Hagmann, Phyllis Kozarsky, David G. Lalloo, Poh-Lian Lim, Calvin Patimeteeporn, Philippe Gautret, Silvia Odolini, François Chappuis, Douglas H. Esposito, for the GeoSentinel Network, Emilie Javelle, Francesco Castelli, Alberto Matteelli, Alice Perignon, Camilla Rothe, Christoph Rapp, Cecile Ficko, Eli Schwartz, Frank von Sonnenburg, Watcharapong Piyaphanee, Udomsak Silachamroon, Andrea Boggild, Perry Van Genderen, Joe Torresi, Mogens Jensenius, Shuzo Kanagawa, Yasuyuki Kato, Cedric Yansouni, Anne McCarthy, Paul Kelly, Bram Goorhuis, Rogelio López-Vélez, Francesco Norman, Marc Mendelson, Peter Vincent, Effrossyni Gkrania-Klotsas, Ben Warne, Denis Malvy, Alexandre Duvignaud, Emanuel Bottieau, Joannes Clerinx, Christina Coyle, Hilmer Àsgeirsson, Hedvig Glans, Patricia Schlagenhauf, Rainer Weber, Frank Mockenhaupt, Gundel Harms-Zwingenberger, Nicholas Beeching, Jan Hajek, Wayne Ghesquiere, Henry Wu, Elizabeth Barnett, Natasha Hockberg, Yukiriro Yoshimura, Natsuo Tachikawa, John Cahill, George McKinley, William Stauffer, Pat Walker, Susan Kuhn, Lin Chen, Daniel Leung, Scott Benson, Carsten Schade Larsen, Christian Wejse, Vanessa Field, Carmelo Licitra, Alena Klochko, Noreen Hynes, Cecilia Perret Perez, Bradley Connor, Holly Murphy, Prativa Pandey, Jean Vincelette, Sapha Barkati, Simin Aysel Florescu, Corneliu Petru Popescu, Lucille Blumberg, Albie De Frey, Susan Anderson, Marc Shaw, AnneMarie Hern, Israel Molina, Johnnie Yates, Hugo Siu, Luis Manuel Valdez, Jean Haulman, David Roesel, Phi Truong Hoang Phu, Sarah Borwein

**Affiliations:** 1 Division of Global Migration and Quarantine, National Center for Emerging and Zoonotic Infectious Diseases, Centers for Disease Control and Prevention, 1600 Clifton Rd NE, Mailstop E03, Atlanta, GA 30329 USA; 2 McGill University Centre for Tropical Diseases, Montreal, Canada; 3 Service des Maladies Infectieuses et Tropicales, GH Pitié-Salpêtrière, Université Pierre et Marie Curie, Paris, France; 4 Department of Global Health and Center for Global Health and Development, Boston University School of Public Health, Boston, MA USA; 5 Tropical Disease Unit, University of Toronto, Toronto, Canada; 6 Victorian Infectious Diseases Service, Royal Melbourne Hospital, Victoria, Australia; 7 School of Public Health and Preventive Medicine, Monash University, Victoria, Australia; 8 Center for Tropical and Travel Medicine, Department of Infectious Diseases, Academic Medical Center, University of Amsterdam, Amsterdam, Netherlands; 9 Steven and Alexandra Cohen Children’s Medical Center of New York, New Hyde Park, New York, NY USA; 10 Department of Medicine, Emory University, Atlanta, GA USA; 11 Liverpool School of Tropical Medicine, Liverpool, UK; 12 Institute of Infectious Diseases and Epidemiology, Tan Tock Seng Hospital, Singapore, Singapore; 13 Lee Kong Chian School of Medicine, Nanyang Technological University, Singapore, Singapore; 14 Unité de Recherche en Maladies Infectieuses et Tropicales Emergentes, Aix Marseille Université, Tropical IHU-Méditerranée Infection, Marseillle, France; 15 Department of Infectious and Tropical Diseases, University of Brescia and Spedali Civili General Hospital, Brescia, Italy; 16 Geneva University Hospital, Geneva, Switzerland

**Keywords:** Malaria, International travel, *Plasmodium* spp, GeoSentinel

## Abstract

**Background:**

More than 30,000 malaria cases are reported annually among international travellers. Despite improvements in malaria control, malaria continues to threaten travellers due to inaccurate perception of risk and sub-optimal pre-travel preparation.

**Methods:**

Records with a confirmed malaria diagnosis after travel from January 2003 to July 2016 were obtained from GeoSentinel, a global surveillance network of travel and tropical medicine providers that monitors travel-related morbidity. Records were excluded if exposure country was missing or unascertainable or if there was a concomitant acute diagnosis unrelated to malaria. Records were analyzed to describe the demographic and clinical characteristics of international travellers with malaria.

**Results:**

There were 5689 travellers included; 325 were children <18 years. More than half (53%) were visiting friends and relatives (VFRs). Most (83%) were exposed in sub-Saharan Africa. The median trip duration was 32 days (interquartile range 20–75); 53% did not have a pre-travel visit. More than half (62%) were hospitalized; children were hospitalized more frequently than adults (73 and 62%, respectively). Ninety-two per cent had a single *Plasmodium* species diagnosis, most frequently *Plasmodium falciparum* (4011; 76%). Travellers with *P. falciparum* were most frequently VFRs (60%). More than 40% of travellers with a trip duration ≤7 days had *Plasmodium vivax*. There were 444 (8%) travellers with severe malaria; 31 children had severe malaria. Twelve travellers died.

**Conclusion:**

Malaria remains a serious threat to international travellers. Efforts must focus on preventive strategies aimed on children and VFRs, and chemoprophylaxis access and preventive measure adherence should be emphasized.

## Background

An estimated 214 million infections and 438,000 deaths attributed to malaria occurred globally in 2015 [1]. Although there have been improvements in global malaria control since 2000, malaria remains a threat to international travellers. Malaria is endemic throughout the tropics and sub-tropics, regions visited by an estimated 25–30 million international travellers annually [2], resulting in an estimated 30,000 travel-related malaria infections [3]. Imported malaria may occur more often along certain travel routes in these areas, and may result in secondary transmission [4] if the infection is brought back to a non-endemic country. Most of the reported 17,471 imported malaria infections among US travellers from 2004 to 2014 were acquired while travelling in Africa; *Plasmodium falciparum* or *Plasmodium vivax* comprised the majority of infections [5]. Most *P. falciparum* exposures cluster in Africa and the Caribbean (Hispaniola) and *P. vivax* exposures occur most frequently in Central America, South America, Asia, and Oceania [4].

Despite the risk of malaria when travelling to an endemic country, and the ability to prevent malaria with proper chemoprophylaxis and mosquito-bite precautions, most international travellers do not have a pre-travel clinical visit with a healthcare provider [6]. However, even among travellers who do receive pre-travel care, some may be non-adherent to chemoprophylaxis due to forgetfulness or medication side-effects, or they may decline to take chemoprophylaxis due to cost, peer advice or low perceived risk [7]. Furthermore, travellers may be prescribed a medication ineffective for the intended travel area [5]. The increasing connectivity of malaria-endemic countries with non-endemic countries via air travel [4, 8], the lack of adequate pre-travel preparation, and personal behaviour that may increase *Anopheles* mosquito exposure [9] may keep malaria as a continued threat to travellers’ health.

The purpose of this analysis is to describe the demographic characteristics, trip details, clinical visit information, and disease attributes of travellers diagnosed with malaria at GeoSentinel Global Surveillance Network sites following travel to malaria-endemic areas.

## Methods

### Data source

GeoSentinel, a global clinician-based sentinel surveillance system of 66 specialized travel and tropical medicine clinics, monitors infectious diseases and other travel-related conditions among international travellers and migrants [10]. It was established in 1995 as a collaboration between the Centers for Disease Control and Prevention (CDC) and the International Society of Travel Medicine. All sites have experience of diagnosing and treating patients with travel-related infectious diseases and contribute systematic surveillance data on patients seen for a travel-related illness [10, 11]. Most sites are affiliated with academic medical centres [11], and are located in 29 countries: 23 sites in Europe, 25 in the USA or Canada, 10 in Asia or Australasia, 3 in Latin America, 2 in Africa, and 2 in the Middle East. Diagnostic confirmation and *Plasmodium* speciation are based on the best reference diagnostic test(s) available in that country, and final diagnosis coding is at a clinician’s discretion. Analysis of GeoSentinel surveillance data has been approved as non-research by a CDC human subjects advisor.

### Inclusion and exclusion criteria

The records of international travellers with a ‘confirmed’ malaria diagnosis (those made by an indisputable diagnostic test) seen at a GeoSentinel site after travel completion from 1 January, 2003 to 30 July, 2016 were examined. Records with a ‘probable’ malaria diagnosis (supported by evidence strong enough to establish presumption but not proof), a missing or unascertainable country of exposure, or an additional acute diagnosis not related to malaria (e.g., dengue) were excluded.

### Data extraction and definitions

Data were extracted on traveller demographics, trip details, clinical visit information (including if a traveller received a pre-travel visit with a healthcare provider), and disease attributes. Malaria was defined as ‘severe’ if the record included a diagnosis code for ‘severe and complicated malaria’ or ‘cerebral malaria’. GeoSentinel designations of ‘severe and complicated malaria’ and ‘cerebral malaria’ are defined using World Health Organization (WHO) classifications [12], and are assigned at a clinician’s discretion.

### Statistical analysis

Data were managed using Microsoft Access. All analyses were descriptive and were performed using SAS version 9.4 (Cary, NC, USA).

## Results

### All travellers with a malaria diagnosis

**Fig. 1:**
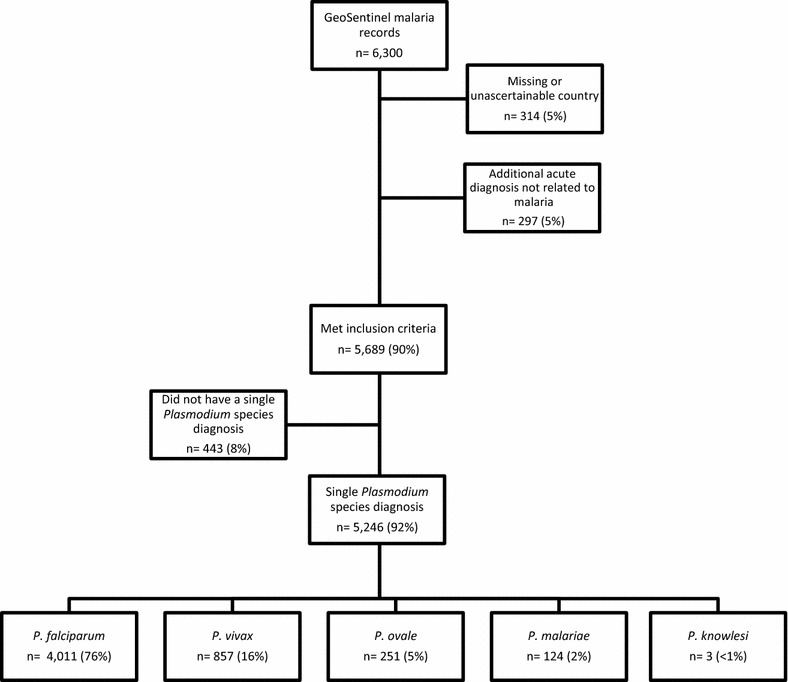
Number of malaria records from GeoSentinel meeting the exclusion and inclusion criteria, and species diagnoses for included cases, January 2003–June 2016

There were 5689 travellers diagnosed with malaria included in the analysis (Fig. 1), including 325 children <18 years of age. The overall median age was 37 years (range 0–88) and 69% were male. Fifty-three per cent (2891 of 5421) of travellers with information available did not have a pre-travel visit (Table 1). More than half (53%) were travellers visiting friends and relatives (VFRs). Eighty per cent of children were VFRs (Table 2); 167 (65%) of the 257 children VFRs were born in the country their parents immigrated to and 231 (90%) travelled to sub-Saharan Africa. Business travel and tourism accounted for 17 and 16% of travel, respectively. Most travellers (83%) were exposed to malaria in sub-Saharan Africa, with malaria infection most frequently occurring after travel to Ghana (9%), Nigeria (8%) or Côte d’Ivoire (8%). The median trip duration was 32 days [interquartile range (IQR) 20–75]; 600 (11%) travellers had a trip duration ≤7 days. Children were hospitalized more frequently than adults (73 and 62% respectively) (Table 2).

**Table 1:** Characteristics of travellers with malaria reported to GeoSentinel, January 2003–June 2016

Characteristic	All travellers (n = 5689)	No species diagnosis (n = 443)	*P. falciparum* (n = 4011)	*P. vivax* (n = 857)	*P. ovale* (n = 251)	*P. malariae* (n = 124)	*P. knowlesi* (n = 3)
Median age, years (range)	37 (0–88)		38 (0–87)	30 (1–88)	36 (2–73)	39 (4–76)	40 (35–52)
Male, n (%)^a^	3918 (69)	304 (69)	2712 (68)	632 (74)	179 (71)	89 (72)	2 (67)
Travellers without pre-travel visit, n (%)^b^	2891 (53)	229 (54)	2173 (57)	373 (47)	69 (29)	46 (39)	1 (50)
Reason for travel, n (%)^c^							
VFRs	3017 (53)	178 (40)	2422 (60)	277 (32)	90 (36)	50 (40)	0 (0)
Business	967 (17)	124 (26)	623 (16)	127 (15)	60 (24)	32 (26)	1 (33)
Tourism	918 (16)	87 (20)	530 (13)	247 (29)	34 (14)	19 (15)	1 (33)
Missionary^d^	514 (9)	49 (11)	330 (8)	74 (9)	40 (16)	20 (16)	1 (33)
Military	184 (3)	8 (2)	44 (1)	108 (13)	23 (9)	1 (1)	0 (0)
Student	77 (1)	6 (1)	50 (1)	18 (2)	1 (<1)	2 (2)	0 (0)
Migrant worker	11 (<1)	0 (0)	7 (<1)	2 (<1)	2 (1)	0 (0)	0 (0)
Planned medical care	4 (<1)	1 (<1)	2 (<1)	1 (<1)	0 (0)	0 (0)	0 (0)
Research	3 (<1)	0 (0)	0 (0)	2 (0.2)	1 (<1)	0 (0)	0 (0)
Region of exposure, n (%)							
Sub-Saharan Africa	4705 (83)	404 (91)	3771 (94)	167 (20)	244 (97)	119 (96)	N/A
South Central Asia	309 (5)	6 (1)	28 (1)	272 (32)	2 (1)	1 (1)	N/A
Southeast Asia	266 (5)	17 (4)	112 (3)	133 (15)	N/A	1 (1)	3 (100)
South America	195 (3)	6 (1)	16 (<1)	171 (20)	1 (<1)	1 (1)	0 (0)
Oceania	96 (2)	3 (1)	13 (<1)	77 (9)	2 (1)	1 (1)	0 (0)
Caribbean	45 (1)	2 (<1)	42 (1)	1 (<1)	0 (0)	0 (0)	0 (0)
North Africa	39 (1)	2 (<1)	26 (1)	8 (1)	2 (1)	1 (1)	0 (0)
Central America	30 (<1)	3 (1)	2 (<1)	25 (3)	0 (0)	0 (0)	0 (0)
Northeast Asia	3 (<1)	0 (0)	0 (0)	3 (<1)	0 (0)	0 (0)	0 (0)
Middle East	1 (<1)	0 (0)	1 (<1)	0 (0)	0 (0)	0 (0)	0 (0)
Median trip duration, days (IQR)	32 (20–75)	31 (17–76)	31 (20–63)	36 (18–101)	56 (22–156)	34 (26–95)	21 (21–52)
Median days between return and presenting to a site (IQR)	11 (6–21)	10 (5–17)	10 (5–16)	30 (13–76)	52 (16–98)	35 (15–64)	21 (2–23)
Hospitalized, n (%)^e^	3523 (62)	345 (78)	2537 (63)	477 (56)	105 (41)	59 (48)	0 (0)

^a^Information was not available for 47 travellers (2 with *P. falciparum*)

^b^Information was not available for 268 travellers (168 with *P. falciparum*, 59 with for *P. vivax*, 14 with *P. ovale*, 5 with *P. malariae*, and 1 with *P. knowlesi*)

^c^Information was not available for 4 travellers (3 with *P. falciparum* and 1 with *P. vivax*)

^d^Includes missionaries, volunteers and aid workers

^e^Information was not available for 1 traveller with *P. falciparum*

**Table 2:** Characteristics of children <18 years of age with malaria reported to GeoSentinel, January 2003–June 2016 (n = 325)

Characteristic	All children (n = 325)	0–5 years^a^ (n = 111)	6–11 years (n = 86)	12–17 years (n = 128)
Median age, years (range)	8 (4–15)	3 (2–4)	8 (7–10)	16 (14–17)
Male, n (%)	185 (57)	54 (49)	49 (57)	83 (65)
Travellers without pre-travel visit, n (%)^b^	166 (53)	56 (51)	45 (54)	65 (53)
Reason for travel, n (%)^c^				
VFR	257 (80)	88 (80)	83 (97)	86 (69)
Tourism	45 (14)	20 (18)	2 (2)	23 (18)
Business	8 (2)	2 (2)	1 (1)	5 (4)
Missionary^d^	6 (2)	1 (1)	0 (0)	5 (4)
Student	6 (2)	0 (0)	0 (0)	6 (5)
Region of exposure, n (%)				
Sub-Saharan Africa	274 (84)^e^	100 (90)	72 (84)	102 (79)
South Central Asia	35 (11)	10 (9)	13 (15)	12 (9)
Southeast Asia	9 (3)	0 (0)	0 (0)	9 (7)
North Africa	2 (1)	1 (1)	0 (0)	1 (1)
Oceania	2 (1)	0 (0)	0 (0)	2 (2)
South America	2 (1)	0 (0)	0 (0)	2 (2)
Caribbean	1 (<1)	0 (0)	1 (1)	0 (0)
*Plasmodium* species, n (%)^f^				
*P. falciparum*	237 (78)	86 (84)	63 (77)	88 (73)
*P. vivax*	46 (15)	10 (10)	14 (17)	22 (18)
*P. ovale*	15 (5)	4 (4)	5 (6)	6 (5)
*P. malariae*	7 (2)	2 (2)	0 (0)	5 (4)
*P. knowlesi*	0 (0)	0 (0)	0 (0)	0 (0)
Median trip duration, days (IQR)	43 (29–90)	42 (28–85)	57 (35–89)	42 (28–108)
Median days between return and presenting to a site (IQR)	12 (6–22)	13 (6–25)	11 (7–20)	12 (6–23)
Hospitalized, n (%)	237 (73)	88 (79)	66 (77)	83 (65)
Severe malaria, n (%)	31 (10)	13 (12)	10 (12)	8 (6)

^a^Three children were <1 year old; all were VFRs who travelled to sub-Saharan Africa and acquired *P. falciparum*

^b^Information was not available for 9 children (1 child 1–5 years of age, 2 children 6–11 years of age, and 6 children 12–17 years of age)

^c^Information was not available for 3 children, all 12–17 years of age

^d^Includes missionaries, volunteers and aid workers

^e^Information was not available for 20 children (9 children 1–5 years of age, 4 children 6–11 years of age, and 7 children 12–17 years of age)

^f^ Of these, 167 (65%) were born in the country their parents immigrated to

### Travellers with a single *Plasmodium* species diagnosis

A single *Plasmodium* species was reported in 5246 (92%) travellers: *Plasmodium falciparum* in 4011 (76%), *Plasmodium vivax* in 857 (16%), *Plasmodium ovale* in 251 (5%), *Plasmodium malariae* in 124 (2%), and *Plasmodium knowlesi* in 3 (<1%) (Table 1).

Among travellers with *P. falciparum*, the most frequent travel reason was VFR (60%), and most were exposed in sub-Saharan Africa (94%) (Table 1). The median number of days between return from travel and presenting at a GeoSentinel site was shortest for *P. falciparum* (10 days, IQR 5–16). Sixty-three per cent of travellers with *P. falciparum* were hospitalized, and 57% (2173 of 3843) of travellers with information available did not have a pre-travel visit; 68% of VFRs with *P. falciparum* did not have a pre-travel visit. Military travellers with *P. falciparum* had the longest median trip duration (107 days, IQR 46–132) and VFRs and tourists with *P. falciparum* had the shortest median trip duration (31 days, IQR 21–58 and 24 days, IQR 14–42 days, respectively).

Travellers acquired vivax malaria most frequently in South Central Asia (32%), with 30% having travelled to either India or Pakistan. *Plasmodium vivax* also caused the majority of malaria in those who travelled to other regions of Asia, the Americas, Oceania, and North Africa. Of 176 military travellers with a single *Plasmodium* species diagnosis, the majority of whom were French, 108 (61%) had *P. vivax*. Among travellers with a single *Plasmodium* species diagnosis, more than 40% with a trip duration ≤7 days and 14% with a trip duration >7 days had *P. vivax*. VFRs who travelled to South Central Asia accounted for 23% of the 228 travellers with *P. vivax* who had a trip duration ≤7 days.

Only 29% (69 of 239) of travellers with information available with *P. ovale* single species infections did not have a pre-travel visit, and sub-Saharan Africa was the most frequent exposure region for travellers with *P. ovale* (97%). The median trip duration and median number of days between returning and visiting a GeoSentinel site were longest for *P. ovale*, at 56 days (IQR 22–156) and 52 days (IQR 16–98), respectively.

### Travellers with a mixed *Plasmodium* infection

Sixty-six (1%) travellers had a mixed infection with *P. falciparum* and *P. vivax* (26 travellers; 39%), *P. falciparum* and *P. ovale* (21 travellers; 32%), *P. falciparum* and *P. malariae* (15 travellers; 23%), *P. vivax* and *P. ovale* (3 travellers; 5%), or *P. vivax* and *P. malariae* (1 traveller; 1%) (Table 3). Most travellers with mixed infections did not receive a pre-travel visit (34 of 58 travellers with information available; 59%), travelled to sub-Saharan Africa (79%), and were VFRs (52%); more than 60% were hospitalized.

**Table 3:** Region of exposure for travellers with a mixed *Plasmodium* infection reported to GeoSentinel, January 2003–June 2016 (n = 66)

*Plasmodium* species	n (%)	Region(s) of exposure (n)
*P. falciparum* and *P. vivax*	26 (39)	Sub-Saharan Africa (15), Southeast Asia (9), South America (1), Central Asia (1)
*P. falciparum* and *P. ovale*	21 (32)	Sub-Saharan Africa (21)
*P. falciparum* and *P. malariae*	15 (23)	Sub-Saharan Africa (14^a^), Oceania (1)
*P. vivax* and *P. ovale*	3 (5)	Sub-Saharan Africa (2), Oceania (1)
*P. vivax* and *P. malariae*	1 (1)	South America (1)
Total	66	

^a^One traveller had severe malaria

### Travellers with severe malaria

Of the 444 (8%) travellers meeting the WHO definition for severe malaria, more than half [246 of 429 (57%) of travellers with information available] did not have a pre-travel visit; most (95%) travelled to sub-Saharan Africa, and the most frequent reason for travel was VFR (42%) (Table 4). There were 124 single *Plasmodium* species diagnoses: 97% were *P. falciparum*, 2% *P. vivax* and 2% *P. malariae*. Thirty-one (10%) of the 325 children in this analysis had severe malaria; children younger than 5 years of age accounted for almost half (42%) of the 31 severe paediatric cases. Fourteen travellers, all adults, had cerebral malaria; all had trip durations >7 days and, per GeoSentinel records, none died. Only five (36%) travellers with cerebral malaria had a species diagnosis recorded; all were *P. falciparum*.

**Table 4:** Characteristics of travellers with severe malaria reported to GeoSentinel, January 2003–June 2016 (n = 444)

Characteristic	
Median age, years (range)	42 (1–88)
Male, n (%)	307 (69)
Travellers without pre-travel visit, n (%) (n = 429)	246 (57)
Region of exposure, n (%)	
Sub-Saharan Africa	420 (95)
Southeast Asia	10 (2)
South Central Asia	4 (1)
Caribbean	3 (1)
North Africa	2 (<1)
Oceania	2 (<1)
Central America	2 (<1)
South America	1 (<1)
Reason for travel, n (%)	
VFR	187 (42)
Business	133 (30)
Tourism	80 (18)
Missionary^a^	33 (7)
Military	7 (2)
Student	3 (1)
Planned medical care	1 (<1)
*Plasmodium* species, n (%) (n = 124)	
*P. falciparum*	120 (97)
*P. vivax*	2 (2)
*P. ovale*	0 (0)
*P. malariae*	2 (2)
Median trip duration, days (IQR)	31 (18–66)
Median days between return and presenting to a site (IQR)	11 (5–16)
Hospitalized, n (%)	402 (91)
Cerebral malaria (n)	14
Deaths (n)	12

^a^Includes missionaries, volunteers and aid workers

Five of 12 travellers who died had a species diagnosis; all were *P. falciparum.* The median age of deceased travellers was 44 years (range 26–66); 55% (6 of 11) of travellers with information available had a pre-travel visit; 92% were exposed in sub-Saharan Africa. Half were business travellers. The median trip duration was 31 days (IQR 15–94).

## Discussion

GeoSentinel, a specialized surveillance network, captured surveillance data from travel medicine facilities around the world and facilitated the description of malaria cases among returned travellers. The WHO’s World Malaria Report 2015 indicated that malaria infections, mostly involving persons living in endemic areas, declined by an estimated 18% from 2000 to 2015 [1]. Efforts to eliminate malaria contributed to this decline, and the WHO’s Global Technical Strategy for Malaria 2016–2030 aspires to continue reducing malaria incidence and mortality by 90% in high-burden countries [1, 13]. Despite improvements in malaria control, imported malaria to the USA has increased since 1973 [5] and stabilized in the UK [14]. These data, together with GeoSentinel data, indicate that malaria continues to pose a health risk to travellers and surveillance of global travel-related malaria infection is essential to travellers’ health, as well as elimination efforts.

Specific traveller groups may more frequently acquire malaria while abroad than others [6]. Males were more frequently diagnosed with malaria than females; males are thought to be at higher risk of malaria [15], possibly from higher travel frequency or increased exposure to mosquito bites from various activities [16]. However, women, children or migrants from high-burden countries may share a similar malaria infection frequency as men, but did not receive care at GeoSentinel surveillance sites. Travellers with malaria more frequently acquired infection in sub-Saharan Africa than other regions. Sub-Saharan Africa remains the most heavily concentrated region for malaria transmission and, likely, traveller exposure [1]. The majority of the malaria burden and malaria deaths worldwide are from 15 African countries [1]; Nigeria and the Democratic Republic of the Congo accounted for approximately 40% of malaria mortality per WHO in recent years [13]. Although the 12 deaths were not exposed to malaria in these two countries, 11 (92%) were exposed in other countries in West or Central Africa. Malaria was also more frequent among VFRs than tourists, business travellers, or other traveller groups, consistent with other published reports [5]. This may be due to a lack of risk awareness among VFRs due to previous residence in a malaria-endemic area, financial barriers that prevent VFRs from obtaining a pre-travel visit or filling prescriptions for prophylactic medications, cultural or language barriers in accessing pre-travel care, adoption of local health-related behaviour during their trip, or travel to areas with high transmission intensity, sometimes for an extended time or with little advance notice [11, 17–19]. A Global TravEpiNet analysis of pre-travel healthcare visits that found VFRs were more likely than non-VFRs to visit malaria-endemic countries [20]. In this analysis, VFRs were most frequently infected with *P. falciparum*; this finding is consistent with surveillance findings from Europe, including the UK, where more than 80% of imported *P. falciparum* malaria was among VFRs [21, 22]; this finding is likely due to a high frequency of travel to West Africa. Preventing malaria (particularly severe malaria caused by *P. falciparum*) among VFRs is best if done proactively; this includes increasing malaria awareness, promoting pre-travel visits and inquiring about future travel plans [23]. Diminishing the burden of imported malaria among travellers to sub-Saharan Africa and VFRs must be a continued focus of malaria prevention efforts. Diminishing this burden will also prevent re-introduction of malaria to locations that have achieved malaria elimination.

This is the largest analysis to date describing characteristics of child travellers with malaria from North America and Europe [24]. One in 10 children treated at GeoSentinel clinics had severe malaria and children <5 years of age accounted for almost half of all severe paediatric cases. Previous studies demonstrated that children account for 15–20% of all imported malaria cases, and approximately 5–10% of children have illness classified as severe, according to WHO criteria [24]; up to one-third of children surviving severe or cerebral malaria will have persistent neurocognitive impairment [25]. Approximately 80% of children diagnosed with malaria were VFRs, and exposure was most common in sub-Saharan Africa. Appropriate preventive care and chemoprophylaxis targeting child VFRs is crucial to help limit travel-related morbidity in this group [24, 26], particularly when travelling to regions where *P. falciparum* predominates. Prevention efforts must include encouraging pre-travel visits for both adults and their children and providing training and education to paediatricians who may be the only healthcare provider a child sees before travelling. Improved identification of potential child travellers and better adherence to preventive measures is imperative to prevent malaria morbidity and mortality in this population.

The majority of severe malaria infections was caused by *P. falciparum* in both adults and children in this analysis, consistent with both WHO reports [12] and US traveller surveillance findings from 2014 demonstrating that 83% of severe cases imported to the USA were *P. falciparum* [5]. Almost all severe malaria infections were acquired in Sub-Saharan Africa, and most frequently among VFRs. A recent multicentre European study describes a similar pattern of geographic exposure and reason for travel among travelers with severe malaria to this current study, but in comparison, report a smaller percentage of travelers ≤18 years with severe malaria (5% vs 7% respectively) [27]. The reason for this difference is unknown, but may be a result of small numbers in each study’s cohort.

Most military travellers with *P. vivax* in this analysis were French, and 58% were exposed to malaria in French Guiana. A possible explanation for this finding is the increasing proportion of vivax malaria found in French Guiana and an increase in French military missions to the country from 1998 to 2008 [28]. However, there is reporting bias, since the Marseille GeoSentinel site contributed the greatest number of records in this analysis, resulting in a large proportion of travellers returning from French-speaking countries.

One in 10 travellers with malaria traveled for 1 week or less, and 40% of short-term travellers with a species diagnosis had *P. vivax*. Short-duration travel is often viewed as low risk and, in some cases, prophylaxis may be declined or not recommended. However, malaria remains a risk even for short stays in an endemic area, and therefore a risk–benefit evaluation of malaria chemoprophylaxis should be considered. A limitation regarding acquiring clinical data from short-term travellers with *P. vivax* or *P. ovale,* is their infection cannot be definitively linked to the most recent travel.

The highest proportion of completed pre-travel visits was among travellers diagnosed with *P. ovale*. Although the reason for this finding is unclear, it may be from greater pre-travel preparation for longer-duration trips, given that travellers with *P. ovale* had the longest median trip duration (55 days). It may also be due to a larger proportion of travellers seeking pre-travel health advice before travelling to Africa, where *P. ovale* is concentrated. It is also possible that compliance with chemoprophylaxis may prevent symptomatic primary infections with *P. ovale*, but not relapses weeks to months later. This latter hypothesis is further supported by the finding that travellers with *P. ovale* had an almost 2-month delay between returning from travel and visiting a GeoSentinel site. However, although both *P. ovale* and *P. vivax* have longer median duration between return and presenting to a GeoSentinel site and both may have a delayed presentation due to hypnozoite reactivation, these findings did not hold for *P. vivax*, suggesting the presence of confounders for this hypothesis.

This analysis of GeoSentinel surveillance data has several limitations. Given the specialized nature of the sites that comprise the surveillance system, these data may not be representative of all travellers with malaria. GeoSentinel is not population-based, so malaria rates and risks cannot be determined. GeoSentinel does not routinely collect information regarding malaria chemoprophylaxis, including the medication taken and compliance; as such, chemoprophylaxis appropriateness or traveller adherence or identify the specific reasons why these travellers became infected cannot be assessed. Nevertheless, given the low proportion of receipt of pre-travel counseling among travellers diagnosed with malaria in this analysis, preparation for travel to malaria-endemic regions was likely inadequate. Severe malaria may not be adequately captured in GeoSentinel, as not all sites have in-patient treatment capabilities and diagnosis relies on clinical identification and provider discretion. Similarly, death is not well-recorded through GeoSentinel surveillance, reflected in the absence of deaths reported among patients with cerebral malaria and the absence of species determination. Data are collected from a single time point, and may not capture a later death, therefore a case fatality rate cannot be calculated. More than 400 travellers did not have *Plasmodium* species information available in GeoSentinel; the entry of a specific *Plasmodium* species was not required prior to 2017, resulting in confirmed malaria without a *Plasmodium* species diagnosis. Also, the lack of diagnostic methodology information collected in GeoSentinel does not allow for independent validation of species diagnoses or mixed-species identifications; although microscopy is the gold standard for malaria diagnosis in most centres [1], mixed-species infections may be overlooked or incorrectly characterized [29, 30]. Despite these limitations, GeoSentinel is the largest surveillance system providing clinical data on travellers, and contributes valuable data on the epidemiology of infectious diseases acquired during international travel and migration.

## Conclusions

Despite general improvements in control programmes, malaria continues to cause serious illness in international travellers, particularly VFRs. By identifying the characteristics of travellers with malaria, prevention efforts may be enhanced, such as proactively asking patients about upcoming travel during routine healthcare contacts, ensuring access to appropriate chemoprophylaxis, increasing risk awareness, and reinforcing the need for prevention measures among higher-risk travellers, including children.
